# Comprehending Adverbs of Doubt and Certainty in Health Communication: A Multidimensional Scaling Approach

**DOI:** 10.3389/fpsyg.2016.00558

**Published:** 2016-05-03

**Authors:** Norman S. Segalowitz, Marina M. Doucerain, Renata F. I. Meuter, Yue Zhao, Julia Hocking, Andrew G. Ryder

**Affiliations:** ^1^Department of Psychology, Concordia UniversityMontreal, QC, Canada; ^2^Faculty of Health, School of Psychology and Counselling, Queensland University of Technology (QUT)Brisbane, QLD, Australia; ^3^Culture and Mental Health Research Unit, Jewish General HospitalMontreal, QC, Canada

**Keywords:** health communication, epistemic adverbs, multidimensional scaling, semantic space, cultural consensus, uncertainty in health communication, physician-patient relations

## Abstract

This research explored the feasibility of using multidimensional scaling (MDS) analysis in novel combination with other techniques to study comprehension of epistemic adverbs expressing doubt and certainty (e.g., *evidently, obviously, probably*) as they relate to health communication in clinical settings. In Study 1, Australian English speakers performed a dissimilarity-rating task with sentence pairs containing the target stimuli, presented as “doctors' opinions.” Ratings were analyzed using a combination of cultural consensus analysis (factor analysis across participants), weighted-data classical-MDS, and cluster analysis. Analyses revealed strong within-community consistency for a 3-dimensional semantic space solution that took into account individual differences, strong statistical acceptability of the MDS results in terms of stress and explained variance, and semantic configurations that were interpretable in terms of linguistic analyses of the target adverbs. The results confirmed the feasibility of using MDS in this context. Study 2 replicated the results with Canadian English speakers on the same task. Semantic analyses and stress decomposition analysis were performed on the Australian and Canadian data sets, revealing similarities and differences between the two groups. Overall, the results support using MDS to study comprehension of words critical for health communication, including in future studies, for example, second language speaking patients and/or practitioners. More broadly, the results indicate that the techniques described should be promising for comprehension studies in many communicative domains, in both clinical settings and beyond, and including those targeting other aspects of language and focusing on comparisons across different speech communities.

## Introduction

How people understand expressions of uncertainty, especially as they relate to discussing medical risk, is a challenging area of study in physician-patient communication (Berry, 2004; Gigerenzer, 2008; Pryer and Hewitt, 2010; Jones, 2013; Pilnick and Zayts, 2014). Consider the following example (adapted from Shah, 2014, p. 801):

[Patient] “It's a lung infection, right, Doc?”
[Physician]“*Perhaps*, … But it could *possibly* be something more serious.”
[Patient] “Why do I need another test? Can't this one tell you what I have?”
[Physician] “We need a more detailed picture, which will *likely* reveal the diagnosis.”

The physician is using the adverbs *perhaps, possibly*, and *likely* to convey the lack of absolute certainty about outcomes (see also Skelton et al., 1999). Such adverbs are inherently vague and may be open to misinterpretation, with potentially serious consequences in situations such as obtaining informed consent or delivering bad news (Babrow et al., 1998; Fox, 2000). Even quantifying uncertainty in terms of probabilities can be open to misinterpretation (Gigerenzer and Edwards, 2003). For this reason, health communication researchers need tools for studying how speakers handle the subtleties of key expressions such as adverbs of uncertainty and doubt. Our goal in this paper is to explore the feasibility of using one particular set of tools for this purpose, with a primary focus on health communication in clinical settings.

We focus on multi-dimensional scaling (MDS) (Kruskal and Wish, 1978; Takane et al., 2009; Armstrong et al., 2014), a class of techniques for analyzing dissimilarity judgments about a set of objects, including words. For example, suppose people are asked to rate how far apart they believe pairs of cities to be (i.e., how “dissimilar” the cities are in location). MDS can translate the judgments into locations in a multidimensional space, where the reported dissimilarities are represented as distances within that space. In this example, it is likely that the cities would be spread out across a 2-dimensional (psychological) space in a pattern corresponding roughly to their geographic distances on a map. Moreover, MDS can be used to discover psychological distances between objects even when the “true” distances and dimensionality of the space are not known, for example, in the case of judgments about word meanings. MDS also reports how well the objects fit into a space of a predetermined number of dimensions. With certain constraints in mind, one can interpret MDS solutions as reflecting how observers mentally organize information about the objects. Here we use MDS to examine how speakers mentally organize meanings associated with uncertainty adverbs relevant to health communication.

Our goal here is to explore some possibilities and limits of using MDS in the context of health communication. Our contribution is twofold. Conceptually, we provide evidence on how native speakers of English in two different English-speaking communities appear to cognitively organize adverbs expressing doubt and certainty. Methodologically, we demonstrate with health-communication relevant stimuli how MDS, when suitably supplemented with other data analysis techniques, can be used to study group-level language phenomena while taking into account inter-individual variability, thus making it possible to study potential group differences in health communication.

In the past, language researchers have used MDS to study how people represent the meanings of *single* words in a variety of domains, including in the context of intercultural comparisons (Hermann and Raybeck, 1981). These include studies of emotion words (Shubert, 1999), verbs, adjectives, and nouns (Arnold, 1971; Segalowitz and de Almeida, 2002; Bybee and Eddington, 2006), household objects (Ameel et al., 2009), politeness terms (Pizziconi, 2007), and—in the health domain—pain descriptors (Janal, 1995). The advantage of using MDS is that one only needs to obtain dissimilarity judgments; participants do not need to explicitly report their understanding as would be the case in direct tests of comprehension. For these reasons, MDS may be useful for studying sources of misunderstanding in health communication contexts. In this study, we examine the basic feasibility of applying MDS to study comprehension of uncertainty adverbs.

The research we report provides a methodological backdrop for future studies of comprehension in health communication in two ways. First, it breaks new ground in being the first to apply MDS to people's understanding of uncertainty adverbs. Second, also for the first time to our knowledge, the task involves presenting stimulus words in explicit health communication contexts by embedding them in carrier sentences (i.e., not as decontextualized, single words). This is important because without explicit contexts participants may create their own frameworks for judging stimuli, including those unrelated to health communication, resulting in unwanted variability in the data. An important methodological aim, therefore, was to see whether using sentences rendered MDS unsuitable for studying comprehension of specific words. The research also looks at the degree of intragroup (within-community) consensus on the meanings of uncertainty adverbs. People belonging to a given language community (say, English speakers) may nevertheless vary in how they use uncertainty adverbs, notwithstanding what a formal linguistic analysis might indicate about how such words are normatively used in that language. Measures of the range of variability in a reference group's use of these words can provide a useful reference point for understanding intragroup variability observed in some other group, for example, second language speakers or speakers of another variety of the language, indicating whether that variability is outside the range normally expected according to the performance of some reference group of speakers.

There are several different types of MDS analyses available and these reflect a tension between simpler group-level solutions and concern for individual differences. Classical MDS analysis (C-MDS; also called two-way MDS) is conducted on a single group-level matrix of proximities (dissimilarity ratings), which represents the aggregated data of all participants' responses. MDS methodologists have argued, however, that such averaging across participants is problematic because it obscures differences in the structure of the data among participants (Ashby et al., 1994). For example, individuals might differ in terms of which dimensions make up their semantic spaces as revealed by MDS analyses. Another possibility is that they might differ in terms of the importance accorded to each dimension. Weighted MDS (W-MDS; also called three-way MDS, or Individual Difference Scaling—INDSCAL) was developed to address these kinds of issues. This approach analyzes an array of proximity matrices (one matrix for each participant) and yields a group solution as well as individual weights indicating how closely a participant's personal solution matches the group solution. W-MDS analyses thus provide measures of the degree to which an individual departs from the central tendency of the group as a whole. Such measures can be useful for determining the generalizability of results across a community of speakers and for examining variations across different language groups.

As mentioned earlier, our ultimate goal is to investigate how people understand uncertainty adverbs in health communication contexts where misunderstandings are likely to arise, especially between first and second language speakers. However, in the studies reported here, we focus only on first language English-speakers in order to explore possible methodological limitations of MDS and the extent of variability or consensus among native speakers. In doing so, we hope it will be possible to identify appropriate ways to extend the methodology to other populations and to draw lessons for the design of future research.

The target adverbs studied here come from Wierzbicka (2006; Chapter 8: *Probably*) and include such words as *apparently, possibly, probably, supposedly*. These were chosen for several reasons. First, there exists a linguistics literature that may shed light on what differentiates one from another (Guimier, 1988; Hoye, 1997; Wierzbicka, 2006). Second, these words are easily embedded in carrier sentences to highlight the health communication context. Third, there exists a literature regarding similar expressions in other languages (e.g., French: Guimier, 1996; Celle, 2009; Mandarin: Lau and Ranyard, 1998; Spanish: Ramón, 2009; Hennemann, 2012).

These words are sometimes referred to as *epistemic adverbs* because, in addition to communicating information about uncertainty, they convey something about the speaker's personal commitment (stance) in relation to that information (Babrow et al., 1998; Gray and Biber, 2012). This stance can include, among other things, level of agreement with what is being asserted, confidence in its truth value, or something about how the information came to be known (see also Guimier, 1988; Hoye, 1997; Wierzbicka, 2006). For example, consider the statement “*This is _____ an allergic reaction,”* where the blank is to be filled with an adverb such as *definitely, possibly, obviously*, or *reportedly*, etc. These adverbs signal that the speaker is affirming the basic situation (presence of an allergic reaction). However, they also signal something about the speaker's stance. *Definitely* and *possibly* convey different degrees of confidence in the speaker's mind, whereas *reportedly* suggests that the knowledge did not come from firsthand experience. The term *obviously* appeals to the listener by suggesting that anyone with the same knowledge as the speaker would logically draw the same conclusion. Thus, native-like understanding of these adverbs involves being able to understand what they convey about the speaker's beliefs and feelings about the information. An important research goal would be to capture how people actually do understand such expressions. MDS may provide insight into such understanding without asking people to explicitly report their knowledge of these nuances.

To obtain MDS-appropriate data, we asked native speakers to provide dissimilarity judgments on pairs of sentences cast as two different “doctors' opinions.” The sentences differed only in the adverb of uncertainty used. In Study 1, we collected data from an Australian sample and investigated the potential and limitations of MDS for use with these stimuli, starting with W-MDS and then turning to cultural consensus theory (Romney et al., 1986), a framework and methodology developed in anthropology to address issues of group consensus and inter-individual variability. In Study 2, we replicated the procedure with a Canadian sample and compared the results across the two English-speaking populations. Finally, we briefly consider the implications of the results for studying language barriers in health communication involving second language speakers.

## Study 1

In this study, we used MDS to investigate how native English-speakers represent epistemic adverbs expressing certainty and doubt in sentences relevant to health communication. We addressed the following questions: First, would W-MDS analysis, when applied to dissimilarity judgments of target words *embedded in sentences*, reveal statistically acceptable solutions (low stress and a high level of explained variance)? The issue here is whether carrier sentences would add noise and mask any underlying structure in response patterns. Second, would W-MDS reveal intragroup consensus within a community of first language English speakers? The issue here was whether MDS with judgments about adverbs could reveal intragroup consistency. Third, would W-MDS analysis reveal interpretable semantic distinctions and would these correspond to those identified in formal linguistic analyses?

### Materials and methods

Participants were 69 English speakers recruited from the student participant pool at a major university in Brisbane, Australia. Those retained for this study reported English as their first or dominant language and rated their English language speaking and listening abilities as “4” or “5” on a 5-point Likert-type scale where “1” indicated *no ability at all* and “5” indicated *fluent ability*. In addition, we excluded participants reporting strong knowledge of another language (abilities reported as ≥3). The initial total sample was 128, of which 92 provided usable data, 74 of these qualified as native or dominant English speakers, and 69 reported no strong knowledge of another language (*M*_age_ = 21.33 years, range = 18–55; 55 females). All participants received course credit for participating.

#### Stimuli

The target words were the following 12 adverbs: *apparently, certainly, clearly, definitely, evidently, likely, obviously, probably, possibly, presumably, reportedly*, and *supposedly*. These were combined to produce 66 different pairs, each adverb occurring 11 times across the sets of pairs. The members of each pair were then embedded in a carrier sentence to express two medical opinions (e.g., *This possibly means you pulled a* muscle; *This presumably means you pulled a muscle*). No adverb appeared in the same carrier sentence more than once. The sentences within a pair were ordered as a First *Opinion* and Second *Opinion*, with each adverb occurring approximately half the time (5 or 6 times out of 11) in First and in Second opinions. For each sentence pair there was a 9-point Likert-type dissimilarity rating scale, ranging from “not different at all” to “extremely different.”

Eight more expressions were used in warm-up and filler trials. These explicitly reflected meanings that could, in theory, separate the sentences in a given pair. Key elements in these sentences were: *I'm sure, I'm positive, from what I've heard, from reports I've seen, it makes sense that, it's logical that, from my experience, from what I know*.

The sentence pairs were organized into a sequence of 98 trials, of which 66 involved target adverb comparisons, 28 involved filler pairs, and four were warm-up trials. To create variety, 33 different carrier sentences were used, each associated with one filler and two adverb expressions (one with only two adverb expressions). Materials were quasi-randomized so that no carrier sentence and no adverb occurred in consecutive trials. Eight more sentence pairs were created for use in instructions, six of which contained filler expressions and two contained adverbs. There were three equally spaced rest breaks, each with three anagram puzzles for distraction.

#### Language background questionnaire (LBQ)

The LBQ is a short questionnaire eliciting basic demographic information about gender, age, knowledge of first and second languages, educational history with respect to known languages, and self-reported proficiency in speaking, listening, reading, and writing skills in each language.

#### The final questionnaire

All materials were placed into SelectSurvey for online access (SelectSurvey, 2014). Order of materials was: (a) Consent Form; (b) Main task—Instructions, 98 sentence pairs, each accompanied by a 9-point rating scale, plus rest pauses; where instructions were to read the pair of medical opinions and rate how different they were, and (c) the LBQ and two catch questions to detect inattentive responding.

### Procedure

Participants answered the questionnaire online from home or other location. This study was carried out in accordance with the recommendations of Concordia University Research Ethics Committee and the Queensland University of Technology's University Human Research Ethics Committee, with informed consent from all participants indicated online.

### Analysis and results

The SelectSurvey data were downloaded and cleaned by removing ineligible and incomplete data, including catch question failures, leaving 69 usable questionnaires. Data from the 66 trials containing the target adverbs were extracted from the larger dataset and a weight matrix was created to handle the missing data (0 for missing responses and 1 for valid responses). The data were submitted to exploratory multidimensional scaling (MDS) using the smacofIndDiff function in the smacof package in R (version 1.7-0; De Leeuw and Mair, 2009; Borg et al., 2013), set for ordinal data and the indscal constraint (Borg et al., 2013). To aid interpretation of the semantic space produced by W-MDS, the MDS configuration of adverbs was then analyzed using hierarchical cluster analysis. Where possible, the more robust median ($\tilde{m}$) and median absolute deviation (*MAD*) are reported instead of the mean and *SD* (see Leys et al., 2013). Means and standard 95% confidence intervals (*95%CI*) are also reported where appropriate. Confidence intervals based on bootstrapped (simulated) data show 2.5th and 97.5th percentiles of the empirical distribution.

#### Data cleaning

We retained data from participants meeting the language eligibility requirements, completing all items, and passing the catch questions. Initial data screening revealed errors in stimulus construction. Four trials (four sentence pairs), involving eight different adverbs, had been accidentally duplicated and four different trials had these same eight adverbs missing from appropriate pairwise combinations. For all participants, the second occurrence of each repeated trial was deleted and the four omitted trials were weighted “0” as prescribed for smacof. As noted in Borg et al. (2013, p. 28), this small amount of missing data should not distort the final outcome in a meaningful way. Thus, for each participant four of 66 data trials were missing—just one data point out of 11 for each of the eight adverbs concerned.

#### Statistical acceptability

In a first pass, we set the number of dimensions to three, the maximum number of stable dimensions to be found with 12 stimuli (Kruskal and Wish, 1978). In a second pass, we set the number of dimensions to two in order to compare the outcome with a 3-dimension solution. For each pass, we used Kruskal's stress (group *Stress-1*), median stress-per-subject (*SPS*), and median squared correlation coefficient (*RSQ*; Popper and Haymann, 1996, p. 167) to evaluate model fit at both group and individual levels. *Stress-1* is a standard MDS “badness of fit” statistic characterizing the group solution, and *SPS* provides a stress value for each participant's solution. *RSQ* is the proportion of explained variance in the scaled data (scaled dissimilarity ratings) by the corresponding distances in the MDS solution (the model distances). *RSQ* values are provided for each individual solution (see Table 1).

**Table 1:** **Model fit results for study 1**.

**Model**	**Group Stress-1 [95% CI]**	**$\tilde{m}$*SPS* (MAD), [95% CI]**	**$\tilde{m}$*RSQ* (MAD), [95% CI]**
**3-DIMENSIONAL SOLUTION**			
Real data (full, *N* = 69)	0.170	0.188 (0.020)	0.479 (0.129)
Random data (1000 iterations)	1.756 [1.740; 1.772]	0.206 [0.203; 0.209]	0.231 [0.209; 0.256]
Real data (trimmed, *N* = 62)	0.166	0.184 (0.021)	0.511 (0.123)
**2-DIMENSIONAL SOLUTION**			
Real data (full, *N* = 69)	0.235	0.257 (0.035)	0.506 (0.155)
Random data (1000 iterations)	2.53 [2.508; 2.555]	0.299 [0.294; 0.303]	0.218 [0.194; 0.241]
Real data (trimmed, *N* = 62)	0.229	0.254 (0.031)	0.519 (0.139)

$\tilde{m}$ *SPS, median stress-per-subject; $\tilde{m}$ RSQ, median R-squared; MAD, median absolute deviation*.

As noted by Giguère (2006), there are no guidelines for interpreting stress values from W-MDS (in contrast to classical MDS for which there exist well-established benchmark values). To assess the statistical acceptability of the model fit values, we resorted to computer simulations. We computed as a comparison measure the stress value that would be obtained if the data had been random and lacked inherent structure (Borg et al., 2013, p. 26). The median model fit values obtained from the real data should be substantially lower (i.e., better fit) than that obtained from a random simulation. For this purpose, we created 1000 arrays of random dissimilarity matrices, each containing 69 random assignments (the number of participants we had) of the 66 inter-adverb dissimilarity measures, for 2- and 3-dimensional (2D, 3D) solutions (we note that these simulations took several weeks to complete on a modern laptop, which may be beyond the computational stamina of most researchers conducting health communication research).

As shown in Table 1, the group *Stress-1* values for 3D and 2D solutions lie well below the corresponding random simulation values and outside the associated 95%*CI* for the random simulations, indicating *Stress-1* values markedly better (lower) than for the random data. Similarly, median stress per subject (*SPS*) values for 3D and 2D solutions are outside the corresponding 95%*CI*s for random simulations, indicating better fit to the data than on simulated random models. Also, median *RSQ* (*R*-*Squared*) values for 3D and 2D solutions are larger than corresponding *RSQ* values for random simulations and outside the associated 95%*CI*s, indicating that the MDS model distances accounted for more variance in the scaled data than in random simulations. Note, however, that the median *RSQ* was slightly higher for the 2D solution, suggesting that a 3D solution did not improve on the explained variance. In contrast, *Stress-1* and median *SPS* values were lower for the 3D than 2D solution, suggesting that a 3D solution may be providing additional useful information. It is, unfortunately, difficult to fully objectively determine the number of dimensions to accept (Borg et al., 2013, pp. 70–74). Therefore, given the exploratory nature of this study, we opted for a 3D solution where feasible, while recognizing its provisional nature. Together these results suggest that using sentences to deliver the stimulus words did not interfere with obtaining statistically acceptable results.

Unfortunately, W-MDS does not offer the MDS equivalent of “winsorizing” participants for dealing with outliers. The group solution reflects the data of all participants equally, even those contributing most to badness of fit. For this reason, and given that configuration weights did not reveal clear subgroups of participants, we re-ran the 3D W-MDS analysis after eliminating the 10% of participants with the lowest *RSQ* values (see Table 1, trimmed sample). As can be seen, model fit increased slightly, especially for *RSQ* values, indicating robust configurations.

#### Individual differences and intra-group consensus

The second goal was to assess intragroup consensus and the extent of individual differences. In addition to a group solution reflecting a pattern characteristic of the entire sample, the W-MDS analyses also yielded configuration weights reflecting individual differences in how much importance each participant attributed to each dimension of the group solution. Weights of 1 on a given dimension indicate that the participant is in perfect agreement with the group solution on that dimension, whereas weights less than 1 indicate that the person attached less importance to the dimension than did the group as a whole and vice versa for weights larger than 1. An individual whose weights on all three dimensions coincided exactly with the group solution would be located at [1, 1, 1] in a 3D space showing participants' weights on each dimension.

**Figure 1:**
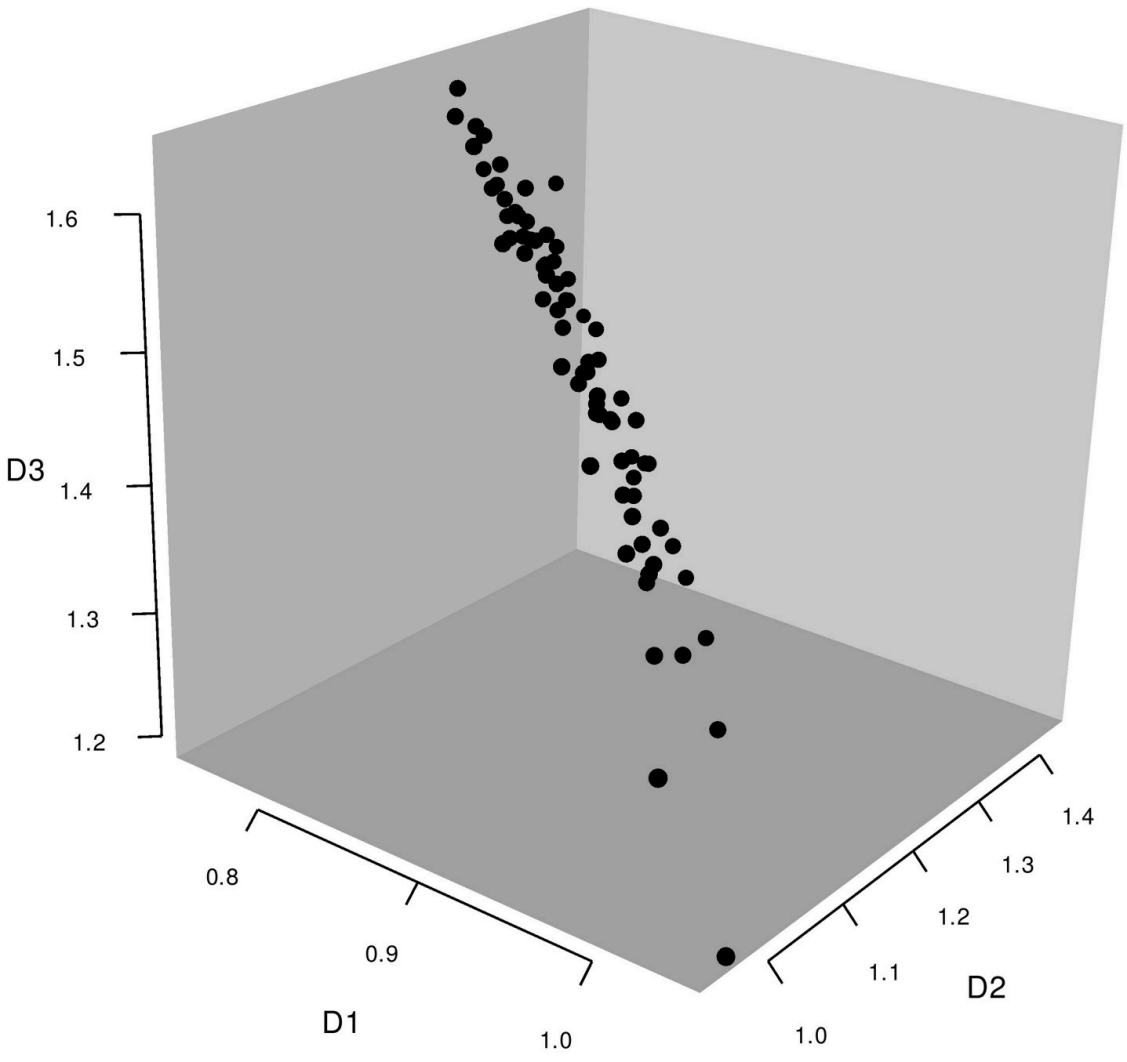
**Individual weights in the 3D W-MDS analysis of epistemic adverbs in the Australian sample**.

When each person's weight for each dimension was plotted in a 3D space (see Figure 1), individual weights showed substantial deviation from the point defined by coordinates [1, 1, 1]. This indicates that most participants departed from the group solution in some way or other. The pattern, however, was not random, which would have indicated idiosyncratic solutions and a lack of intragroup consistency. Also, the pattern of deviations did not yield identifiable clusters, such as some points near [1, 1, 1] and others clustering elsewhere, which would have indicated subsets of the population systematically attaching different levels of significance to the dimensions. For example, in a classical W-MDS study of body parts by Jacobowitz (1973), reported by Takane et al. (1977), individual weights formed two clear clusters corresponding to adults and to children. Here, rather, weights were distributed along a relatively clear flat plane, suggesting that although the data were not random, neither were there clear subgroups within the sample. Thus, individual deviation from the group solution may reflect noise more than systematic variability. This outcome is consistent with the idea of general intragroup consensus. Also supporting this interpretation, the geometric distance between individual configuration weights (points in the 3D weight space) and the coordinate point [1, 1, 1] correlated well (*r* = −0.56) with individual *RSQ* values (proportion of variance in the scaled data accounted for by the MDS model), indicating that the more a person's perceptions approached the group solution, the greater the accounted-for variance in their pattern of responses. This geometric distance also correlated moderately with *SPS* values (*r* = 0.43), especially for dimension 1 (D1), the correlations for the three dimensions being −0.55, 0.27, and 0.46 respectively. The evidence, therefore, is generally consistent with the idea that members of this Australian group of English-speakers interpreted adverbs of uncertainty in similar ways, and that departure from the group solution indicated noise rather meaningful individual variation.

A potential problem with the analyses reported so far is that the group solution given by W-MDS accords equal weight to all participants, despite indexing individual variability in weights associated with each dimension. W-MDS might, therefore, be better suited to addressing individual differences as a principal goal than it is to providing information about the group's consensus after taking into account individual differences. Indeed, most studies using a W-MDS approach then focus on understanding what characteristics of individuals can account for variations in emphasis given to the different dimensions, as in Takane et al.'s (1977) examination of adults vs. children. Our goal here was different; we sought to find the intersubjective normative cultural representation of epistemic adverbs among Australians (i.e., the dominant representation members of this cultural group generally believe to be widely shared in the culture Wan et al., 2007). In that sense, a C-MDS (classic MDS) was conceptually closer to our goal. C-MDS is also easier from a practical point of view to implement. There exist well-established benchmark values to estimate model fit when using C-MDS, whereas with W-MDS we had to resort to computer simulations to interpret the stress values we obtained. As noted earlier, these simulations took several weeks to complete on a modern laptop, a serious limitation. However, we share other MDS researchers' (e.g., Ruette and Speelman, 2014) concerns about simply aggregating data by computing mean ratings across all participants. Rather, we are interested in “meaningful aggregation.” Now, when we try to estimate a cultural intersubjective norm, we have to take into account that participants will vary in their knowledge of this norm and so we need an aggregation method that takes into account individual differences in this knowledge. In short, our interest was in a practical method to study intragroup consensus that takes into account individual variation in knowledge of this consensus. We turned therefore to cultural consensus theory (Romney et al., 1986) as an alternative way to address the second research question. As presented below, we used a cultural consensus approach supplemented by classical MDS (C-MDS) to analyze data aggregated across participants. This allowed us to enter individual difference results from the cultural consensus analysis into the C-MDS analysis.

Developed in anthropology, the cultural consensus framework distinguishes two sources of variability: cultural variability (the existence of different “subcultures” or clusters of people) and variability in individual differences in knowledge competence (knowledge of the core, culturally dominant information) (Borgatti and Halgin, 2011). Further, this framework postulates that estimates of participants' knowledge of a cultural intersubjective norm can be estimated from levels of agreement among people. This approach can help establish whether there is one core, consensual semantic representation for the adverbs within the speaker sample. Second, provided no subgroups are identified, a cultural consensus approach allows one to assess how much each person conforms to or knows about the consensual representation. Measures of individual differences in this knowledge can then be used to more precisely characterize the group consensus by taking into account these individual differences when using C-MDS.

Methodologically, consensus analysis uses factor analysis on participants as units of analysis rather than items or scales as is typically the case (Weller, 2007). The factor loadings so derived are conceptualized as “competence scores,” that is, measures of the extent to which participants “know” the cultural consensus. These scores can be used in two ways. First, they provide a metric to eliminate from analysis those participants who depart excessively from the group consensus. Second, they can be used as weights in the computation of the group aggregated data that is then analyzed through C-MDS: i.e., instead of all participants contributing equally to the group average, those with higher factor loadings (indexing greater “knowledge” of the consensus) contribute to the group average more strongly than those with lower loadings. In short, C-MDS paired with cultural consensus analysis allows us to assess a cultural intersubjective norm while taking into account individual variation in knowledge of this consensus—which is our goal here. To our knowledge, this study is the first one using this step-wise approach.

We conducted a consensus analysis by performing a minimum residuals factor analysis (Weller, 2007) on participants using the fa function in the psych package (v. 1.5.6; Revelle, 2015) in R. Factor analysis requires a rows-to-columns ratio of at least 5:1 (Gorusch, 1983), with higher ratios being preferable. Thus, with 66 similarity judgments (rows), our factor analysis should include no more than 13 participants at a time. We had 69 participants. We resolved this by factor analyzing a randomly selected subset of 10 participants at a time (a ratio of almost 7:1), repeating the procedure 1000 times and retaining median values from these 1000 repeats (this simulation took only minutes). We used participants' factor loadings (cultural competence scores) as weights in computing a group-level weighted average dissimilarity matrix, where participants with higher factor loadings contributed more than participants with lower loadings. This single matrix of aggregated data was then analyzed using the SmacofSym function (with the ordinal constraint) of the smacof package in R, which performs a C-MDS analysis.

#### Results of the consensus analysis

The existence of a group consensus was supported by a ratio of first-to-second factor eigenvalues >3.0 (following standard recommendations, Weller, 2007). This indicates that splitting participants into a second “group” (or factor, given this was a factor analysis of participants) accounted for proportionally little additional variance compared to keeping only one “group” (or factor). We obtained a ratio of 7.73 (ratio of first: second eigenvalues = 3.69:0.48), well above the conventional recommendation of a 3:1 ratio. The factor loadings on the 1-factor solution provided individual cultural competence scores indexing the degree to which each person's data correlated well with the factor (Weller, 2007). The median competence score was 0.62 (*MAD* = 0.14), above the recommended 0.50 average (Weller, 2007), indicating that there was a single consensual representation of the target adverbs. As a rule of thumb, competence scores below 0.30 are considered to indicate considerable departure from consensus (Weller, 2007), a value also cited as a lenient rule-of-thumb cutoff value in exploratory factor analysis (more precisely, 0.32; Tabachnick and Fidell, 2001). In this sample, eight participants had competence scores under 0.30 and so were eliminated. We then computed a group-level weighted average dissimilarity matrix using competence scores as weights.

#### Statistical acceptability of the weighted-data C-MDS results

Table 2 reports model fit values for both 2D and 3D solutions. For comparison purposes, we report fit values for both weighted and unweighted (i.e., with no adjustment by factor loadings, using simple mean aggregation) analyses. As can be seen, using weighted data improved the model fit over unweighted data, the weighted data yielding higher *RSQ* and lower *Stress-1* values, supporting the use of consensus analysis. All results reported next refer to the weighted data analyses. According to Kruskal and Wish (1978), *Stress-1* values below 0.05 are considered excellent, between 0.05 and 0.10 are good, between 0.10 and 0.20 are fair, and above 0.20 are poor. For *RSQ*, the minimum acceptable value is 0.60. In this sample, model fit indices for the weighted analysis favored a 3D solution (*Stress-1* = 0.075, *RSQ* = 0.867) over a 2D solution (*Stress-1* = 0.112, *RSQ* = 0.862). The semantic analyses reported next, therefore, are based on the 3D solution.

**Table 2:** **Model Fit Results for Study 1 with the Australian sample using Classical MDS with and without weighted data derived from cultural consensus analysis (see text for details)**.

**Model**	**Australian Sample**	
	***Stress-1***	***RSQ***
**3-DIMENSIONAL SOLUTION**		
Weighted data	0.075	0.867
Unweighted data	0.068	0.842
**2-DIMENSIONAL SOLUTION**		
Weighted data	0.112	0.862
Unweighted data	0.123	0.855

*RSQ, R-squared*.

#### Semantic analysis

**Figure 2:**
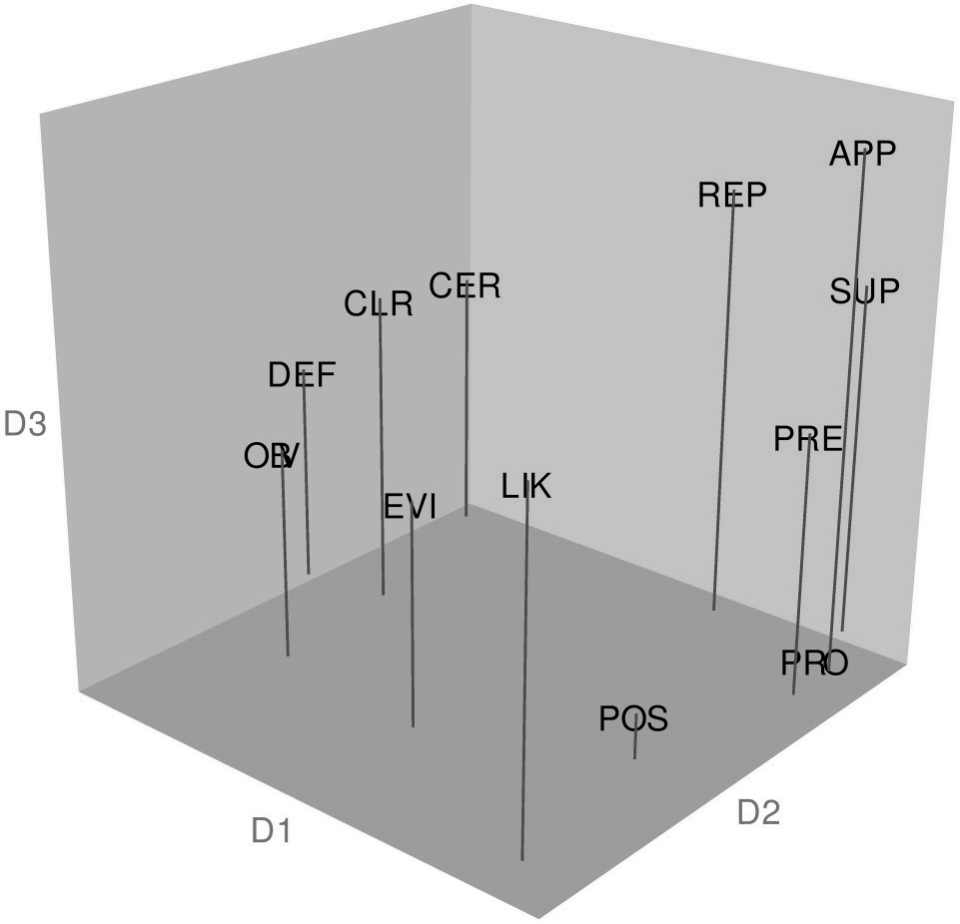
**Configuration of target adverbs in the Australian sample and the results of a hierarchical cluster analysis of the coordinates from the MDS spatial configuration**. The abbreviations for the 12 target adverbs are: APP, apparently; CER, certainly; CLR, clearly; DEF, definitely; EVI, evidently; LIK, likely; OBV, obviously; POS, possibly; PRE, presumably; PRO, probably; REP, reportedly; and SUP, supposedly. The analysis revealed the following clusters: Cluster 1 (Jaccard similarity value = 0.960): apparently, presumably, reportedly, supposedly; Cluster 2 (Jaccard similarity value = 0.856): certainly, clearly, definitely; Cluster 3 (Jaccard similarity value = 0.795): evidently, likely, obviously; Cluster 4 (Jaccard similarity value = 0.865): possibly, probably.

Our third research question was whether MDS analysis would yield interpretable semantic distinctions. Figure 2, shows the 3D group solution based on the consensus/MDS analysis. Interpreting an MDS configuration involves subjective and qualitative approaches that take into account the existing literature (see Borg et al., 2013). This is because specific dimensions serving as plot axes can be arbitrarily rotated (including obliquely) and so there is no guarantee that the dimensions will be meaningful. To aid interpretation, the coordinates for each adverb, taken from the group solution in the weighted-data C-MDS analysis, were submitted to hierarchical cluster analysis, using the R package “fpc” (flexible procedures for clustering; Hennig, 2015) with clustermethod=hclustCBI, method=ward.D2, k=4, and 100 bootstrap replications (for other examples combining MDS with cluster analysis, see McLaughlin et al., 1991; Leonard and Ashley, 2012). Figure 2 also reports the clustering patterns that emerged from this analysis and their Jaccard similarity values (see Hennig, 2007). A Jaccard similarity value = 0.75 is considered to indicate a “valid, stable cluster” and = 0.85 indicates a “highly stable” cluster (Hennig, 2015, p. 30).

Figure 2 shows that D1 reflects a contrast between adverbs expressing higher levels of confidence (*certainly, clearly*, and *definitely* on the left side of the plot along D1, members of cluster 2) vs. lower levels of confidence (*probably* and *possibly*, on the right side of D1, members of cluster 4). The cluster analysis also reveals a set of words (cluster 1) referring to the speaker's information source—*reportedly, apparently, presumably*, and *supposedly*, all of which convey that the information source is at some “distance” from the speaker. Cluster 3 contains the words *evidently, likely*, and *obviously* which reflect a “close” information source, indicating that were the listener in possession of the same background information as the speaker then he or she would logically draw the same conclusion.

### Discussion

The results answered the three research questions in the affirmative. First, MDS analysis proved feasible for use with dissimilarity ratings on adverbs embedded in carrier sentences. This feasibility was supported by the high level of variance accounted for by the MDS solution (*RSQ* values) and the low level of stress (*Stress-1* values below that of randomized data). This result occurred in both W-MDS and in weighted data C-MDS analyses. Importantly, the results were stronger with the weighted data C-MDS analysis (*RSQ* values >0.80). These results not only extend the use of MDS to epistemic adverbs, a semantic domain not before studied this way, but they also indicate one can use carrier sentences to ensure stimuli are understood as intended. Second, there was evidence for intragroup consistency (consensus), seen in the strong fit when weights derived from the consensus analysis were used with the C-MDS analysis.

Finally, the meaning structure revealed by the MDS solution was interpretable and corresponded to analyses found in the linguistics literature. For example, a major contrast emerged between adverbs expressing higher confidence (Wierzbicka, 2006) or conviction (Hoye, 1997) vs. lower confidence or conviction (cluster 2 vs. 4). Interestingly, Wierzbicka also includes *evidently* in the “confident” category whereas our data and Hoye's (1997) analysis do not (more on *evidently* later in Study 2). The data also revealed a group of words—cluster 1: *apparently, presumably, reportedly, supposedly*—conveying a personal stance about the speaker's knowledge source, namely, the knowledge is from evidence that does not come from direct experience. Note that evidentials—adverbs referring to knowledge supported by evidence—can also convey a level of conviction or of doubt. Hoye (1997) points out, for example, that *apparently* is a lower conviction adverb than are *clearly* and *obviously* because it conveys doubt in the sense that “what is said can only be understood as the speaker's interpretation rather than as a personal assessment of a particular state of affairs” (p. 192). Our data support this distinction between adverbs explicitly indicating that knowledge is indirect knowledge—*reportedly, supposedly, apparently* (knowledge through hearsay), and *presumably* (a conclusion that goes beyond what the speaker could directly know; Wierzbicka, 2006, p. 257)—vs. those indicating either more direct knowledge based on some form of thinking about the matter (cluster 3: *evidently, likely*, and *obviously*) or not indicating any information about source (cluster 4: *possibly*, and *probably*). Furthermore, while *evidently, likely*, and *obviously* (cluster 3) do appear to express high confidence, that confidence is based only on thinking about the matter (logical deduction based on other knowledge) and thus contrasts with *certainly, clearly*, and *definitely* (cluster 2; Wierzbicka, 2006, pp. 274–275). What we see here, then, is a range of subtle variations in how people can use adverbs of uncertainty to not only convey certainty or doubt, but also to express level of confidence, source of the information, manner by which one came to the opinion, or some combination of these. While linguistic analyses can reveal how languages—in principle—provide different ways to package these nuances, the augmented MDS analysis conducted here was able to empirically demonstrate that English-speakers are indeed guided by knowledge of such nuances, thereby further supporting the feasibility of using MDS to study how people mentally represent adverbs of uncertainty.

**Figure 3:**
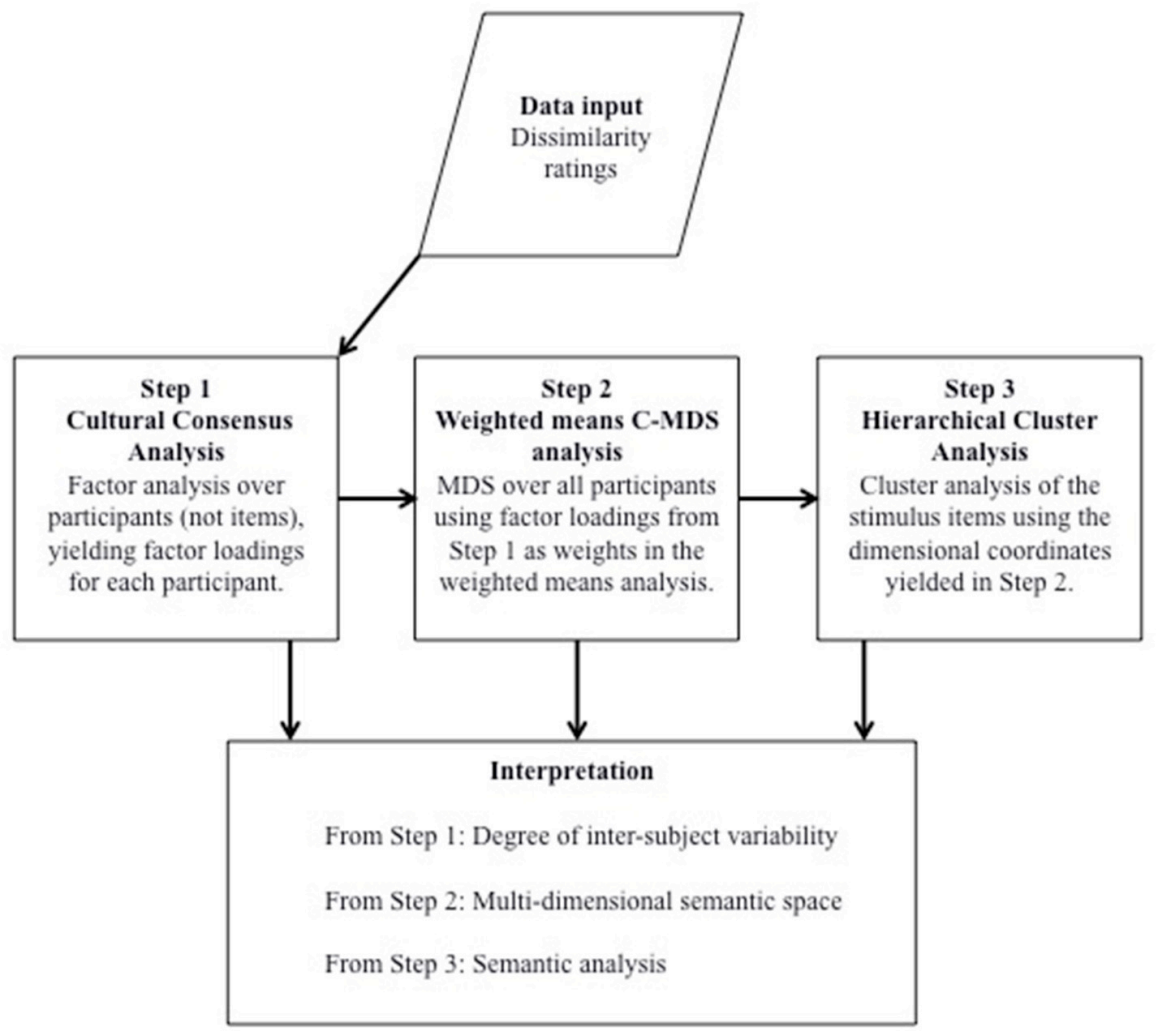
**Summary of the steps involved in the augmented classic multi-dimensional scaling (C-MDS) analysis employed in this study (see text for details)**.

In summary, the results of this study demonstrated the feasibility of employing MDS together with a form of cultural consensus analysis and cluster analysis to reveal meaningful patterns in the way a group of speakers understand health-communication relevant epistemic adverbs (Figure 3 summarizes the analysis steps). Given the success of this application of MDS to dissimilarity ratings obtained from one English-speaking community, it would be valuable to see if the results can be broadly replicated with another English-speaking community and whether subtle differences between the two communities can also be discerned in the data. That was the goal of the next study.

## Study 2

This study builds on Study 1 by replicating the data collection and analysis procedures with a new sample of native English-speakers from Canada. There were two main research questions. First, with a new sample would the MDS approach yield overall acceptability of results in terms of low Stress-1 values, high *RSQ* values, intragroup consensus, and semantic interpretability? Second, would it be possible to extend the analysis to compare the Australian and Canadian result patterns?

### Materials and methods

Participants were native speakers of English recruited from the participant pool at a major university in Montreal, Canada. The initial sample numbered 160, of which 69 qualified as dominant in English. As in Study 1, we excluded those reporting strong knowledge of another language. The final sample retained consisted of 19 participants (*M*_age_ = 23.05 years, range = 18–40; 15 females). All received course credit for participating.

### Materials

The materials were identical to those described in Study 1.

### Procedure

Data were collected in parallel with Study 1, using identical procedures.

### Results

Data were cleaned and prepared for analysis as in Study 1. For the first research question on the overall statistical acceptability of the results, we conducted a three-phase analysis: consensus analysis (factor analysis over participants), followed by C-MDS analysis on the single matrix of aggregated data (once with weighted data and once without), and semantic analysis using hierarchical cluster analysis (based on the coordinates from the C-MDS solution). For the second question on comparing results from the Australian and Canadian samples we used semantic analysis and a stress decomposition procedure as described below.

#### Overall statistical acceptability of results

The three phases of the analysis for addressing the overall statistical acceptability of the results are now described in turn.

#### Consensus analysis

As described in Study 1, we conducted a minimum residuals factor analysis (Weller, 2007). We factor analyzed a randomly selected subset of 10 participants from the sample of 19, repeating this procedure 1000 times. Each participant's median factor loading across the 1000 repetitions was interpreted as a cultural competence score, that is, as a measure of that person's degree of consensus with the group solution. These loadings were used to weight each participant's data in the C-MDS analysis applied to the data (see next section).

The consensus analysis (factor analysis) yielded a ratio of first-to-second eigenvalues of 5.77 where 3.0 is the desired minimum (eigenvalues were 3.59 and 0.62, respectively), and strong cultural competence scores (consensus with the group, operationalized as factor loadings on the single factor solution) with a median of 0.62 (*MAD* = 0.173), where 0.50 is the desired minimum (Weller, 2007). These results indicate that there was a consensual representation among the Canadian participants. There was, however, also variability across participants in their loadings, with scores ranging from 0.055 to 0.775, including scores under 0.30 for three participants. We eliminated these three participants when computing the group-level weighted average dissimilarity matrix using weighted data based on the loadings. These results indicate overall consensus across the group of Canadian English-speakers in their responses to the target adverbs.

#### C-MDS analyses

Table 3 reports model fit values for 2D and 3D solutions obtained with the C-MDS analysis using SmacofSym, using unweighted data and weighted data based on the factor loadings obtained in the consensus analysis. As can be seen in Table 3, both the unweighted and weighted analyses yielded statistically acceptable results, namely low *Stress-1* values (all < 0.11) and high *RSQ* values (all >0.88). These values indicate fits as good as or better than that obtained in Study 1 (compare with Table 2). The results justified 3D solutions from both the weighted and unweighted C-MDS analyses. As can be seen in Table 3, the weighted data analyses yielded superior fits of the model data to the dissimilarity ratings compared to the unweighted analyses (lower *Stress-1* values and higher *RSQ* values). For this reason, the analyses presented below are based on the 3D solution with the weighted data analysis.

**Table 3:** **Model Fit Results for Study 2 with the Canadian sample using Classical MDS (C-MDS) with and without weighted data derived from cultural consensus analysis (see text for details)**.

**Model**	**Canadian Sample**	
	***Stress-1***	***RSQ***
**3-DIMENSIONAL SOLUTION**		
Weighted data	0.046	0.941
Unweighted data	0.053	0.919
**2-DIMENSIONAL SOLUTION**		
Weighted data	0.088	0.892
Unweighted data	0.109	0.886

*RSQ, R-squared*.

#### Semantic analysis

**Figure 4:**
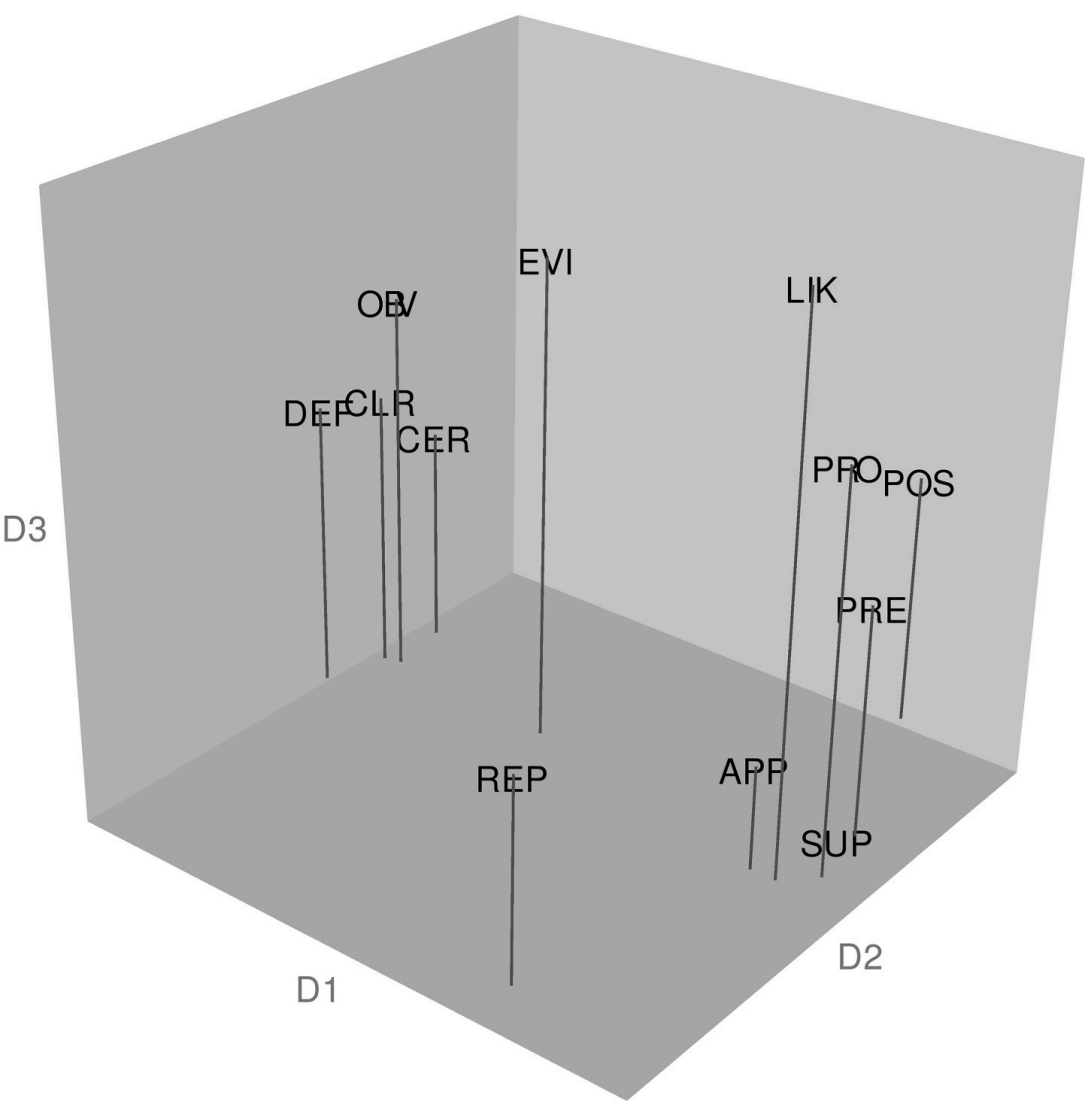
**Configuration of target adverbs in the Canadian sample and the results of a hierarchical cluster analysis of the coordinates from the MDS spatial configuration**. The abbreviations for the 12 target adverbs are: APP, apparently; CER, certainly; CLR, clearly; DEF, definitely; EVI, evidently; LIK, likely; OBV, obviously; POS, possibly; PRE, presumably; PRO, probably; REP, reportedly; and SUP, supposedly. The analysis revealed the following clusters: Cluster 1 (Jaccard similarity value = 0.823): apparently, possibly, presumably, supposedly; Cluster 2 (Jaccard similarity value = 0.869): certainly, clearly, definitely, obviously; Cluster 3 (Jaccard similarity value = 0.814): evidently, likely, probably; Cluster 4 (Jaccard similarity value = 0.635): reportedly.

Figure 4 shows the 3D configuration yielded by the weighted C-MDS analysis and reports the results of hierarchical cluster analysis using the fpc package, based on the coordinate values from the C-MDS analysis, and showing both the cluster patterns and the corresponding stability measures. The results reveal a cluster of “confident” adverbs—*certainly, clearly, definitely*, and *obviously* (cluster 2, Jaccard similarity value = 0.869). The analysis also reveals a cluster generally referring to the speaker's information source—*apparently, possibly, presumably*, and *supposedly* (cluster 1, Jaccard similarity value = 0.823), conveying information that could be seen as having its source at some distance from the speaker. Cluster 3 (Jaccard similarity value = 0.814) consists of the words *evidently, likely*, and *probably*, which convey confidence but not great certainty. The final “cluster” (cluster 4) has only *reportedly* as its member and a low Jaccard similarity value (0.635), the interpretation of which Hennig (2015) indicates would be difficult to make and “highly doubtful” (p. 30).

Overall, the results indicate successful replication of Study 1 with a new sample, in terms of strong community consensus, low *Stress-1*, high *RSQ*, and generally stable and interpretable semantic outcomes. The semantic analysis revealed some differences compared to the semantic outcomes reported in Study 1, and these are considered in the next section.

## Comparison of the Canadian and Australian samples

The two steps for comparing the Australian and Canadian results are now described in turn.

### Semantic analysis

As revealed above and in Study 1, each set of speakers differentiated a similar set of “confident” adverbs (cluster 2) from the rest. Interestingly, the Canadian sample included *obviously* in its cluster 2 whereas the Australian sample did not; nevertheless, *obviously* was located near cluster 2 in that sample's 3D semantic space. This distinction (cluster 2 vs. the rest) seems to reflect D1. If one orders all items along D1 in the respective 3D solutions, the correlation between the Australian and Canadian sets is very strong—Spearman *rho* = 0.91 (*p* < 0.0001). However, the first four items on this dimension (the higher confidence level items) appear to be more tightly clustered in the Canadian set than in the Australian set (see the respective MDS spaces). Both Canadian and Australian participants also seem to group together three adverbs expressing indirect knowledge (*presumably, apparently, supposedly*) but differ in terms of a fourth adverb in this cluster—*possibly* in the Canadian and *reportedly* in the Australian data (compare cluster 1 from each sample). In the Canadian set *reportedly* did not enter into a valid, stable cluster. Despite these differences, the two groups appear to generally resemble each other on these two aspects of underlying meaning of uncertainty adverbs.

### Stress decomposition analysis

**Figure 5:**
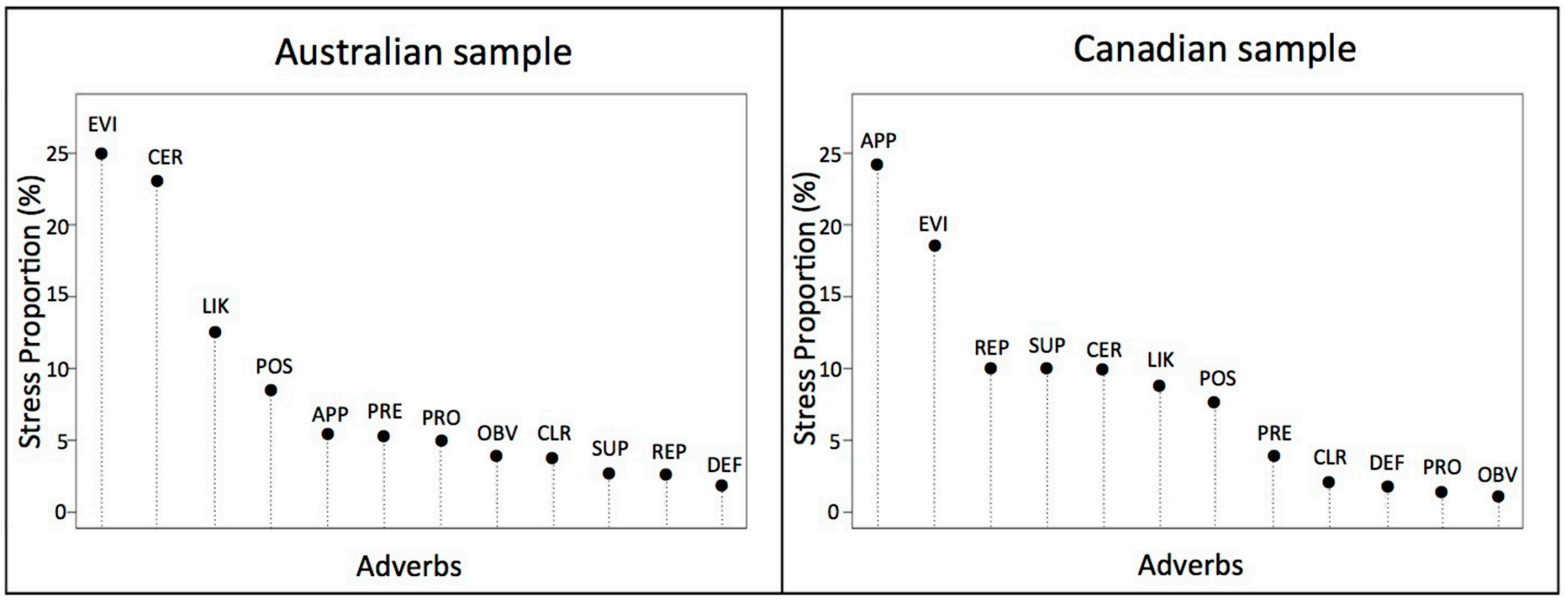
**Stress decomposition per adverb (percent contribution to overall stress by each adverb) in each of the Australian and Canadian data sets**. The abbreviations for the 12 target adverbs are: APP, apparently; CER, certainly; CLR, clearly; DEF, definitely; EVI, evidently; LIK, likely; OBV, obviously; POS, possibly; PRE, presumably; PRO, probably; REP, reportedly; and SUP, supposedly.

We also compared the two data sets by decomposing *Stress-1* values, looking at each adverb's stress-per-point (*SPP*) as a percent of total stress. These decomposition values reflect how easily each adverb fit into the solution space (Figure 5). In each data set, 10 of the 12 adverbs accounted for 13% or less of the stress, with many accounting for 5% or less. The similarity of values indicates that most adverbs contributed to the overall picture in much the same way, and their low values indicate the participants had little difficulty making the ratings. The latter is not surprising, given that all were native speakers of English. For each data set, however, two adverbs did not fit as easily as the other 10. In the Australian sample *certainly* and *evidently* were outliers (*SPP* = 23 and 25%, respectively) and in the Canadian sample *apparently* and *evidently* were outliers (*SPP* = 19 and 24%, respectively).

### Discussion

This study replicated the basic results of Study 1 with a new sample of participants, showing that classical-MDS, when combined with consensus analysis and complemented by hierarchical cluster analysis, can reveal statistically acceptable and linguistically meaningful results regarding the comprehension of epistemic adverbs presented in sentence contexts. In addition, the results showed that it was possible to compare results from two different speech communities. Goodness-of-fit values, semantic space solutions, stress decomposition analysis and cluster stability measures all indicated strong similarities between the groups as well as some differences.

One interesting group similarity is that the same word—the adverb *evidently*—turned out to be an outlier for both speech communities in terms of its high SPP value (how poorly it fit into the spatial solution), as seen in Figure 5. Wierzbicka (2006, p. 271) observed that *evidently* indicates that the source of a speaker's confidence derives from “thinking rather than knowledge” and conveys the message that “if other people thought about this evidence, they would have come to the same conclusion.” Hoye (1997, p. 192), somewhat differently, argues that *evidently* signals an element of doubt because it indicates a conclusion based on interpretation and not directly experienced knowledge. Guimier (1988) makes a similar point. Thus, there appear to be at least two nuances underlying the word *evidently*, one related to an indirect knowledge source and the other to an element of doubt. Interestingly, in the Australian sample *evidently* appears in a cluster along with *obviously* whereas in the Canadian sample it occurs together with *probably*. These appear to reflect the two different interpretations of the word identified by the linguists cited above, suggesting that Australian speakers emphasize the indirect knowledge element of its meaning whereas Canadian speakers emphasize the doubt element of its meaning. Unfortunately it was beyond the scope of this study to explore this speaker-group difference more deeply but, now that this difference has been revealed in speakers' comprehension of sentences using the word, future research on this difference seems merited. Finally, it should be noted that, of the 12 adverbs, *evidently* occurs the least frequently in the English language (Davies, 2008). Perhaps, then, the high SPP-values for *evidently* reflect response inconsistency arising from these different considerations.

With respect to group differences, the adverb *apparently* had a high *SPP-*value in the Canadian sample but not the Australian, whereas the reverse was true for *certainly*. Wierzbicka (2006, pp. 277–278) points out that *apparently* has three possible interpretations, one related to *it appears that*, another indicating hearsay as the information source, and a third conveying a noncommittal stance regarding the truth of the statement. All three could have been evoked by the stimulus sentences used in this study. Perhaps the two communities differ in how likely each meaning comes to mind. For example, if Canadian participants typically accessed any or all three whereas the Australian participants focused on only one or two, this could account for the asymmetry in *SPP* values. Similarly, the adverb *certainly* has multiple nuances. Wierzbicka (2006) indicates it can convey assent or agreement, especially in sentence initial position and also “the speaker's full (not merely subjective) certainty (p. 286)” about the information. Hoye (1997) also distinguishes between possibility and necessity, a distinction that may have affected interpretations of *certainly* in the present research. For example, expressions conveying possibility (*this could certainly mean …*) occurred 4/11 times, those conveying necessity (*this certainly means …*) occurred 6/11 times, and one example might have conveyed assent (in sentence initial position, *Certainly, …*). Again, there might have been speech community differences in how these alternative interpretations affected responding.

The important conclusion here is that methodologically it was possible to reveal similarities and differences between the two speech communities in their comprehension of these epistemic adverbs, indicating the potential utility of MDS for future study of language barriers in health communication, including where second language speakers are involved.

## General discussion

Our main goal was to explore the feasibility of using MDS to study word comprehension relevant to language barriers in health communication. For this purpose, we focused on adverbs of doubt and certainty and on data from two different communities of first language speakers of English. The logic of this approach was that if MDS did not work well with first language speakers it could not be expected to work well with other groups, such as second language speakers. In addition, it was important to discover if presenting target words in sentences that highlight the health communication context would in some way undermine the use of MDS. We also wanted to explore obtaining group-based solutions that took into account individual variability. The results of the two studies reported here supported the feasibility of using MDS. In particular, the results demonstrated a three-step approach to be useful. First, the dissimilarity rating data were submitted to a cultural consensus analysis (factor analysis over participants, not items) to obtain factor loadings indicating how much each person performed in accord with the group as a whole. Second, these factor loadings were used to weight the dissimilarity ratings in a classical multidimensional scaling (C-MDS) analysis where the weighted ratings were averaged over participants to yield a single data matrix. Third, the coordinates for each adverb in the semantic space generated by the C-MDS analysis were used in a hierarchical cluster analysis to reveal underlying patterns of stable clusters that helped to interpret the structure within the semantic space. This three-phase analysis yielded solutions that strongly met statistical acceptability criteria in MDS research, it generated an interpretable semantic space for the target adverbs, and it demonstrated the possibility of making comparisons across speech communities. This three-phase approach proved to be more feasible than an individual differences W-MDS (INDSCAL) approach and made it possible to avoid some of the limitations of W-MDS analyses, including the need to run lengthy simulations with random data.

In sum, the results provided a backdrop supporting the use of MDS, supplemented by cultural consensus and cluster analyses, for future studies. The techniques described here should be of special value for the study of language barriers due to cultural differences between patient and physician or to language discordance, that is, where patient and physician speak different first languages. Language discordant physician-patient encounters are becoming increasingly frequent because of the growing mobility of populations, both in terms of the linguistic diversity of patients (immigrants, national linguistic minorities) and growing reliance everywhere on health practitioners coming from other countries (Jacobs et al., 2006; Segalowitz and Kehayia, 2011). Such intercultural and language discordant situations may be especially vulnerable to miscommunication, with obvious possible serious consequences given that health issues are involved. The techniques described here have theoretical applications for probing the nature of misunderstanding that can arise in health communication. They also can be of value as a tool for assessing individuals and groups in the context of practical measures taken to provide language and/or cultural sensitivity training for health practitioners.

## Author contributions

NS, RM, YZ, JH, and AR were responsible for the original conception and design of this research and for the data acquisition. MD had the lead role in conducting the statistical analyses, working together with NS and JH. NS and MD were primarily responsible for writing the manuscript but all co-authors contributed substantially to the final version. All were involved in the interpretation of results.

## Funding

The authors would like to thank the Australian Research Council for a Discovery Project grant (DP130104164) awarded to RM, Cindy Gallois, NS, and AR, which supported this research. We also thank Health Canada for an earlier grant to McGill University's Training and Retention of Health Professionals (TRHP) project and subaward to NS and AR of the Health-Care Access for Linguistic Minorities (H-CALM) research team in support of earlier work leading up to this study.

### Conflict of interest statement

The authors declare that the research was conducted in the absence of any commercial or financial relationships that could be construed as a potential conflict of interest.
